# Speed-related increases in plantar tissue stress without affected-side laterality in individuals with unilateral shoulder dislocation

**DOI:** 10.3389/fpubh.2026.1916758

**Published:** 2026-08-27

**Authors:** Zhenghui Lu, Liufeng Xiao, Peiyao Liang, Molong Chen, Lin Guo, Lin Ma, Dianwei Li

**Affiliations:** 1 Department of Orthopedics/Sports Medicine Center, State Key Laboratory of Trauma, Burn and Combined Injury, Southwest Hospital, Army Medical University, Chongqing, China; 2 Faculty of Engineering, University of Pannonia, Veszprém, Hungary; 3 Department of Physical Therapy, University of British Columbia, Vancouver, BC, Canada

**Keywords:** affected-side laterality, finite element analysis, foot biomechanics, gait analysis, plantar fascia stress, plantar pressure, unilateral shoulder dislocation, walking speed

## Abstract

**Introduction:**

Unilateral shoulder dislocation may be accompanied by altered upper-limb use and trunk-control strategies; however, whether individuals with unilateral shoulder dislocation exhibit affected-side laterality in foot mechanics during walking remains unclear. This study compared the affected-side laterality in plantar external loading and internal foot tissue stresses in individuals with unilateral shoulder dislocation during 5 km/h and 7 km/h treadmill walking conditions, and further examined whether faster walking was associated with changes in this laterality.

**Methods:**

Twelve participants completed walking trials at two speeds. Peak forces in the rearfoot and forefoot regions were extracted, and the effects of walking speed, affected-side status, and dominant-foot status were analyzed using foot-level linear mixed-effects models. A simplified peak-force-based finite element analysis was used to estimate relative stress responses in key foot soft tissues during loading-response- and push-off-related loading conditions.

**Results:**

The results showed that increased walking speed significantly elevated forefoot peak force (*p* = 0.005) and was associated with higher stresses in forefoot soft tissues (*p* = 0.025) and the plantar fascia (*p* = 0.003). In contrast, both plantar external loads and internal tissue stresses remained highly symmetrical between the ipsilateral and contralateral feet relative to the affected shoulder, and increasing speed did not amplify affected-side laterality.

**Conclusion:**

These findings indicate that, within this cohort, faster walking was associated with higher push-off-related plantar tissue stresses, whereas affected-side laterality relative to the dislocated shoulder was not evident. Because no healthy control group or longitudinal pre-injury comparison was included, the results should be interpreted as within-cohort mechanical responses rather than evidence that shoulder dislocation itself increases plantar tissue stress.

## 1 Introduction

Arm swing during walking is not merely an “ancillary” movement; rather, through its coordination with trunk rotation, it contributes to the regulation of whole-body angular momentum, thereby reducing trunk oscillations and enhancing gait stability (1, 2). Previous studies have demonstrated that restricting or altering arm swing patterns can markedly influence trunk kinematics and dynamic stability control, and may induce a reorganization of lower-limb kinematics as well as ground reaction force distributions (1, 3). In individuals with unilateral shoulder dislocation or instability, pain, sensations of instability, and concerns about recurrent dislocation frequently elicit kinesiophobia (4), which in turn leads to reduced arm swing amplitude or asymmetric arm swing on the affected side and promotes compensatory adjustments in trunk rotation strategies (5). Within the theoretical framework of coupled control, such alterations in upper-limb and trunk control may further propagate distally to the lower limbs, resulting in systematic adjustments in bilateral foot loading during the loading response and push-off phases, thereby giving rise to distal compensatory manifestations (6).

Walking speed is considered an important external challenge capable of eliciting and amplifying compensatory mechanisms (7). Under level-ground walking at a preferred speed, the neuromuscular system is typically able to distribute potential compensations across multiple joints through inter-joint coordination, thereby masking bilateral loading differences at the global level. As walking speed progressively approaches the critical transition between walking and running, the demands for propulsion and stability increase concurrently, and reliance on arm swing and trunk control becomes substantially greater. Under these conditions, latent asymmetric control strategies are more likely to emerge in lower-limb load distribution (8). From the perspective of functional gait phases, the loading response primarily emphasizes postural stability and impact attenuation, rendering the rearfoot particularly sensitive to changes in stability-related control strategies. In contrast, the push-off phase is dominated by propulsive function, during which forefoot loading increases markedly with walking speed. Accordingly, examining peak forces in the rearfoot and forefoot regions under preferred-speed and critical-speed walking conditions may provide a more targeted characterization of bilateral load redistribution patterns in response to altered stability and propulsion demands.

Although previous studies have reported the effects of shoulder instability or upper-limb functional limitations on gait parameters and plantar pressure patterns (9–11), these analyses have largely remained at the level of global metrics or spatiotemporal parameters. Quantitative descriptions centered on laterality are scarce, and walking speed has seldom been incorporated as an external challenge. More importantly, findings based solely on plantar external loading are insufficient to elucidate the mechanical costs of these alterations at the level of internal foot tissues. Finite element models of the foot allow estimation of internal mechanical responses, such as plantar fascia tension and stress distributions within forefoot and heel soft tissues, under prescribed external loading conditions (12, 13), thereby providing mechanistic evidence to assess whether distal compensations translate into a potential risk of tissue overload. Recent treadmill-walking studies have shown that plantar pressure variability and asymmetry can provide useful information regarding gait stability and fatigue-related loading changes (14). In addition, foot biomechanical modeling studies have demonstrated the value of combining external loading measures with internal mechanical estimates to interpret foot function and intervention effects (15). Accordingly, integrating laterality analyses of region-specific plantar peak forces with evaluations of internal foot tissue mechanics may advance the investigation of distal compensation beyond surface-level phenotypes toward a more explanatory biomechanical mechanism-based perspective.

Based on the above considerations, this study aimed to compare affected-side laterality of peak forces in the rearfoot and forefoot regions during preferred-speed and critical-speed walking under laboratory conditions, and to further examine whether walking speed, as an external challenge, modulates this laterality. In addition, experimentally obtained region-specific peak forces were applied as simplified loading conditions to a foot finite element (FE) model to evaluate differences in soft tissue stresses associated with changes in walking speed and lateralized loading, thereby exploring potential internal tissue-level mechanical mechanisms. We specifically distinguished between lateralized distal compensation, defined as differences between the foot ipsilateral and contralateral to the affected shoulder, and bilaterally concordant load modulation, defined as an overall increase in loading in both feet without evident side-to-side differences. On this basis, we formulated the following hypotheses: (1) detectable affected-side laterality would be present during level-ground walking; (2) as walking speed increased from a preferred to a critical level, this laterality would be amplified or exhibit a directional shift; and (3) finite element analyses would reveal a corresponding redistribution or increase in mechanical loading of key foot soft tissues under affected-side or higher-speed conditions.

## 2 Materials and methods

### 2.1 Participants

A total of 30 individuals with unilateral shoulder dislocation were initially recruited for this study. During testing, 18 participants were unable to complete the fast treadmill-walking condition with a stable walking pattern and were therefore excluded from the final analysis. Walking data from 12 participants who successfully completed both the 5 km/h and 7 km/h treadmill-walking conditions were ultimately included. All included participants had no history of lower-limb injury or surgery affecting gait within the preceding 6 months, and none presented with neurological disorders, evident balance impairments, or other musculoskeletal conditions that could influence walking patterns.

Basic anthropometric characteristics, including age, height, and body mass, were recorded for all participants. Foot dominance was determined by self-report, defined as the foot preferentially used in daily activities such as kicking a ball, and was considered together with affected-side information in subsequent grouping analyses. Detailed demographic characteristics of the participants are presented in Table 1.

**Table 1:** Participant characteristics (Mean ± SD).

Sex	*n*	Age (years)	Height (m)	Body mass (kg)	Affected side vs. dominant side
Male	10	25.42 ± 5.50	1.77 ± 0.04	75.29 ± 15.46	4 same, 6 opposite
Female	2	30.50 ± 3.50	1.70 ± 0.10	72.00 ± 17.00	1 same, 1 opposite

### 2.2 Gait testing

**Figure 1:**
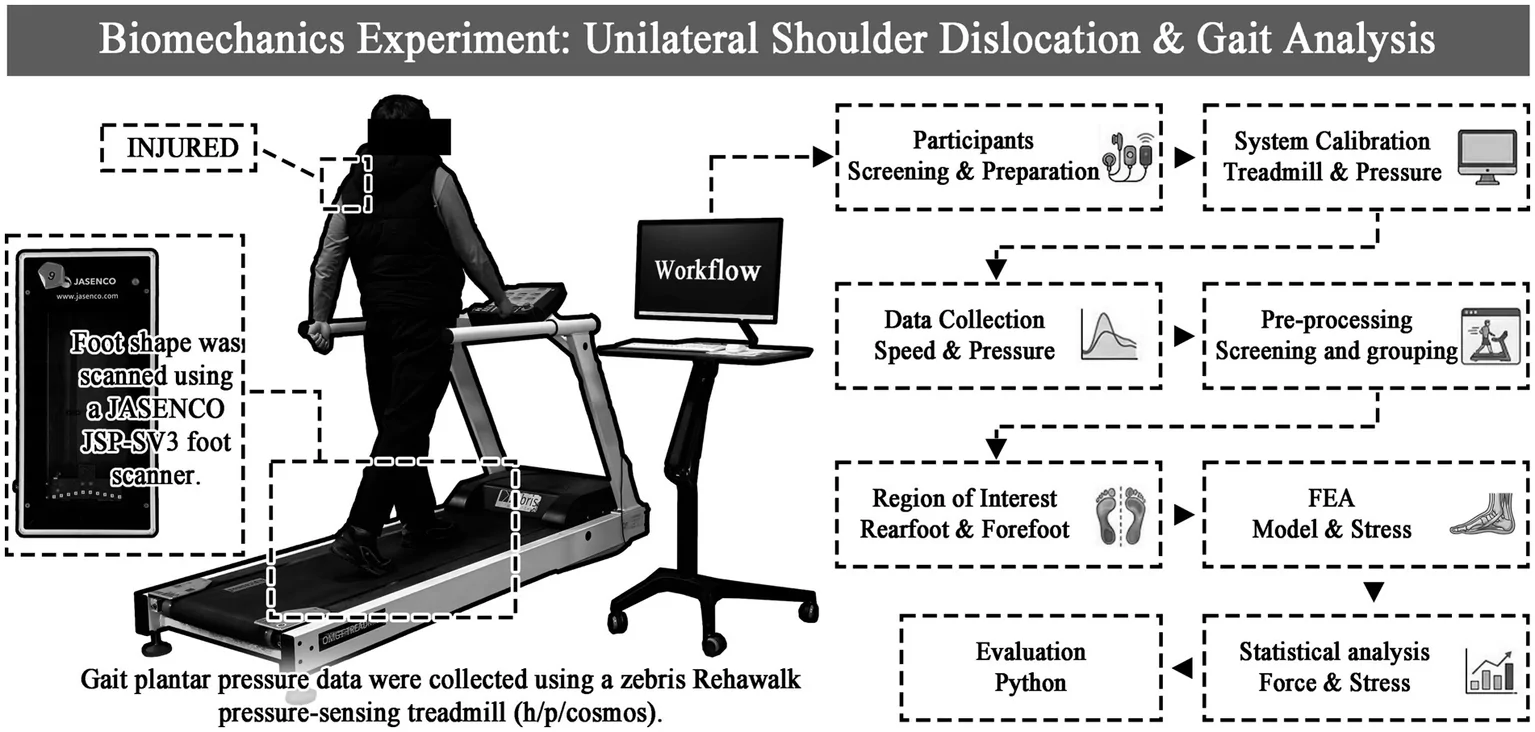
Experimental setup and workflow for plantar-pressure testing and finite element analysis in participants with unilateral shoulder dislocation. Heel soft-tissue stress was extracted under the loading-response-related rearfoot loading condition, whereas forefoot soft-tissue stress and plantar fascia stress were extracted under the push-off-related forefoot loading condition.

As illustrated in Figure 1, gait testing was conducted on an instrumented treadmill system equipped with plantar pressure measurement capabilities (FDM-THPL-S-3i, Zebris, Germany). Each participant completed treadmill-walking trials at two fixed speeds: 5 km/h and 7 km/h. The 5 km/h condition was used as a moderate walking-speed condition. The 7 km/h condition was used as a fixed fast-walking challenge to increase propulsion- and stability-related mechanical demands. Participants were instructed to maintain a continuous walking pattern and not to transition into running. Trials were stopped or repeated if the participant could not maintain stable treadmill walking.

Under each speed condition, participants first performed a brief familiarization walk to minimize the influence of initial gait instability on the measurements. Once a steady-state gait pattern was achieved, plantar pressure data were continuously recorded for 5 min.

### 2.3 Foot finite element model

**Figure 2:**
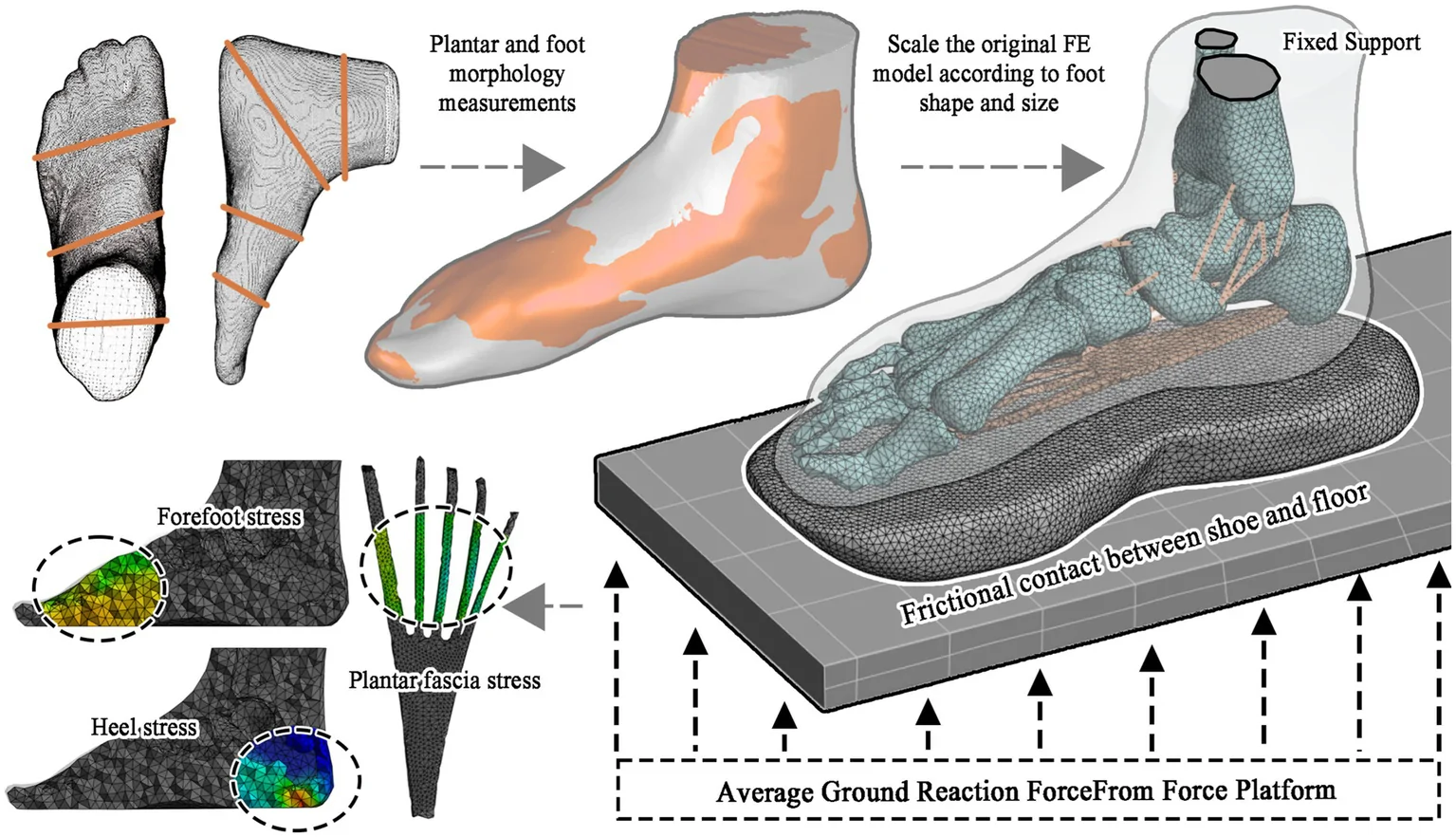
Workflow of foot finite element modeling and stress analysis based on plantar and foot morphology measurements.

The foot FE model was constructed based on a previously published and validated foot model (16). To account for inter-individual variability, the baseline model was geometrically scaled according to each participant’s foot morphology parameters (17), thereby generating morphology-scaled foot models while preserving proportional relationships among key anatomical structures (Figure 2). After geometric scaling, each model was checked for unrealistic element distortion, initial contact penetration, contact stability, boundary-condition consistency, and convergence of the simplified FE solution. The meshing strategy followed that of the original model, with osseous structures and soft tissues represented using solid elements, and ligaments modeled as tension-only link elements. The baseline model has undergone systematic validation in prior studies; because no modifications were made to the material properties or fundamental structural configuration in the present work, no additional experimental validation was performed.

Material properties were assigned in accordance with previously published literature and the specifications of the baseline model (16, 18). Briefly, bone tissue was approximated as a linear elastic material, plantar soft tissues were modeled using a constitutive formulation suitable for large-deformation behavior, and the plantar fascia was represented as a tension-bearing structure with corresponding elastic parameters (Table 2). Frictional contact was defined both between the plantar surface and the footwear, and between the footwear and the ground, with a friction coefficient of 0.5 to capture interaction characteristics at the contact interfaces. To ensure stable load transfer, the contact definitions and initial gap treatment followed those implemented in the baseline model.

**Table 2:** The element types and material properties of the FE model.

Component	Type	Property	Density (kg/m^3^)	Poisson’s ratio (*ν*)
Bone (34, 35)	3D-tetrahedra	E: 17000 MPa	1990	0.3
Cartilage		E: 1 MPa	–	0.4
Soft Tissue (36)		E: 0.15 MPa	950	0.45
Floor		E: 14200 MPa	1,000	0.1
Footwear		E: 2.74 MPa	2,300	0.35
Ligaments (anterior talofibular, anterior tibiofibular, calcaneofibular, posterior talofibular, tibiocalcaneal, tibiotalar anterior/posterior, spring ligament, and tibionavicular) (37)	Two-node tension-only truss	Hyperelastic Material Cross-section area: 1.5–5.91 mm^2^	1,000	-
Other Ligaments (34, 35)		Linear Elastic Material E: 264.8 MPa Cross-section area: 1.784 mm^2^	1,000	0.4
Plantar Fascia (34, 35, 38)	3D-tetrahedra	Hyperelastic Material	1,000	-

Finite element analyses were conducted as simplified peak-force-based loading analyses under two loading conditions corresponding to loading-response-related rearfoot loading and push-off-related forefoot loading. For each participant, the mean peak forces measured in the rearfoot and forefoot regions under the 5 km/h and 7 km/h walking conditions were applied to the model as equivalent vertical loads. These loads were imposed in the form of equivalent vertical ground reaction forces acting on the inferior surface of the ground plate, representing the dominant loading characteristics associated with stability control during initial contact and propulsion during push-off in gait. Proximal constraints were applied at the superior cross-sections of the tibia and fibula to restrict global rigid-body motion, while allowing physiologically reasonable foot deformation and the development of internal stress distributions under external loading.

### 2.4 Outcome measures and statistical analysis

In this study, the foot was treated as the unit of analysis. Observations obtained for each foot under different walking speed conditions were organized and modeled accordingly. Each foot-level observation was labeled based on affected-side status (ipsilateral or contralateral to the affected shoulder) and dominance status (dominant or non-dominant foot), yielding four combinatorial conditions: dominant foot on the affected side, non-dominant foot on the affected side, dominant foot on the contralateral side, and non-dominant foot on the contralateral side. It should be noted that, for an individual participant, the dominant and non-dominant foot cannot simultaneously occur on the affected side, and the same applies to the contralateral side. However, at the group level, all four combinations were represented across the sample, allowing assessment of the main effects of affected-side status and dominance, as well as their potential interaction effects on foot mechanical responses.

Following completion of gait testing, region-specific peak forces were extracted step by step within the steady-state walking interval. For each foot, mean rearfoot peak force and mean forefoot peak force were calculated under preferred-speed and critical-speed conditions, and values were further normalized to body weight. Finite element outcome measures focused on loading related to stability during initial contact and propulsion during push-off. Specifically, peak stress in heel soft tissues was extracted under the loading response condition, whereas forefoot soft tissue stress and plantar fascia stress were obtained under the push-off condition. These metrics were used to evaluate relative changes in tissue-level mechanical demand across walking speeds and side conditions.

To quantify bilateral differences and their modulation by walking speed, laterality was characterized using a symmetry index (SI) (19):


$$\mathrm{SI}=\frac{X_{A}-X_{B}}{(X_{A}+X_{B})/2}\times 100\%$$

𝑋 denotes the output variable. For affected-side laterality, A represents the foot ipsilateral to the affected shoulder and B represents the contralateral foot; for dominant-foot laterality, A represents the dominant foot and B represents the non-dominant foot.

This study aimed to determine whether distal compensation associated with shoulder dislocation manifests as systematic differences between the foot ipsilateral to the affected shoulder and the contralateral foot, and to further evaluate whether such differences are modulated by walking speed as an external challenge and by foot dominance. Statistical inference was performed using linear mixed-effects models, with each mechanical outcome modeled at the foot level. Walking speed, affected-side status (IPSI vs. CON), dominance status (DOM vs. NDOM), and their interaction terms were specified as fixed effects, while participant was included as a random intercept to account for within-subject correlations arising from repeated measurements.

After model fitting, *post hoc* comparisons based on estimated marginal means were conducted, with particular emphasis on differences between the ipsilateral and contralateral feet relative to the affected shoulder, as well as on the interaction between walking speed and affected-side status to assess whether speed modulated affected-side laterality. In addition, interactions between affected-side status and dominance status, as well as the three-way interaction (speed × affected-side status × dominance status), were examined to evaluate the potential modulatory role of dominance on distal compensation patterns. When multiple outcomes and multiple comparisons were involved, Holm or false discovery rate corrections were applied as appropriate. The significance level was set at *α* = 0.05, and effect estimates with corresponding 95% confidence intervals were reported to reflect both effect magnitude and uncertainty. All statistical analyses were performed in a Python environment. Model assumptions were assessed by visual inspection of residual distributions, residual-versus-fitted plots, and standardized residuals. Potential influential observations were checked to ensure that the main findings were not driven by extreme values. Random-slope structures were considered; however, given the limited sample size and the repeated foot-level data structure, more complex random-slope models were unstable or overparameterized. Therefore, participant-level random intercepts were retained as the primary random-effects structure.

## 3 Results

### 3.1 Affected-side effects of plantar external loading and modulation by walking speed and dominance

Model-estimated marginal means of plantar external loading under different walking speeds and foot attribute combinations are presented in Table 3. Under both preferred-speed and critical-speed conditions, differences in rearfoot and forefoot peak forces between the IPSI and CON feet relative to the affected shoulder were generally small, and no clear separation was observed across different foot attribute combinations. Increasing walking speed was primarily associated with an overall elevation in forefoot peak force, rather than a discernible redistribution of load toward the affected side.

**Table 3:** Model-estimated marginal means (EMMs) of rearfoot and forefoot peak forces under different walking speeds and foot attribute combinations.

Combination	Speed (km/h)	Rearfoot		Forefoot	
		Peak force (N)	Normalized (N/kg)	Peak force (N)	Normalized (N/kg)
CON–DOM	5	538 (501, 575)	6.82 (6.49, 7.15)	693 (590, 795)	8.73 (7.69, 9.77)
CON–NDOM		505 (470, 540)	6.52 (6.07, 6.98)	675 (530, 820)	8.61 (7.17, 10.05)
IPSI–DOM		475 (412, 538)	6.10 (5.52, 6.68)	700 (569, 830)	8.94 (7.73, 10.15)
IPSI–NDOM		537 (500, 574)	6.81 (6.53, 7.08)	688 (606, 770)	8.68 (7.92, 9.43)
CON–DOM	7	604 (546, 661)	7.65 (7.03, 8.27)	906 (806, 1,006)	11.56 (10.11, 13.01)
CON–NDOM		523 (394, 652)	6.64 (5.45, 7.83)	847 (782, 912)	10.95 (9.96, 11.94)
IPSI–DOM		512 (363, 660)	6.45 (5.20, 7.70)	840 (787, 892)	10.92 (9.52, 12.33)
IPSI–NDOM		617 (555, 679)	7.81 (7.19, 8.42)	885 (795, 974)	11.32 (9.85, 12.78)

IPSI = foot ipsilateral to the affected shoulder; CON = contralateral foot; DOM = dominant foot; NDOM = non-dominant foot.

To quantify the direction and magnitude of affected-side laterality, symmetry indices (SIs) were further calculated based on the model-estimated marginal means (Table 4). The results indicated that, during the loading response phase, rearfoot peak force exhibited a mild negative laterality under preferred-speed walking (SI = −3.35%), whereas this laterality was markedly attenuated and approached zero under critical-speed conditions (SI = −0.24%). During the push-off phase, forefoot peak force SIs were close to zero under both speed conditions, ranging from −1.21 to 1.60%.

**Table 4:** Affected-side laterality derived from model-estimated marginal means (SI, %, IPSI vs. CON).

Outcome	5 km/h	7 km/h
Rearfoot peak force (N/kg)	−3.35 (−6.95, 0.25)	−0.24 (−4.38, 3.89)
Forefoot peak force (N/kg)	1.60 (−1.59, 4.80)	−1.21 (−4.96, 2.54)

Statistical inference results from the foot-level linear mixed-effects models are summarized in Table 5. For body-mass–normalized rearfoot peak force, the main effect of affected-side status did not reach statistical significance (*p* = 0.297). The main effect of walking speed was also non-significant, and the modulatory effect of speed on affected-side differences likewise failed to reach statistical significance (*p* = 0.202). Notably, the interaction between affected-side status and dominance status exhibited a marginal trend (*p* = 0.075), suggesting that affected-side load distribution may be modulated by functional foot dominance; however, this tendency did not constitute robust statistical evidence given the current sample size. For body-mass–normalized forefoot peak force, the main effect of walking speed was statistically significant (*p* = 0.005), indicating a substantial increase in propulsion-related external loading under critical-speed conditions. In contrast, the main effect of affected-side status was not significant (*p* = 0.937), nor was the interaction between walking speed and affected-side status (*p* = 0.817). Interaction terms involving dominance status also failed to reach statistical significance (all *p* > 0.6).

**Table 5:** Foot-level mixed-effects model results for rearfoot and forefoot peak forces.

Fixed effect	Rearfoot		Forefoot	
	β (95% CI)	p	β (95% CI)	p
Speed (7 vs. 5 km/h)	0.12 (−1.13, 1.36)	0.853	2.34 (0.71, 3.96)	0.005
IPSI vs. CON	0.28 (−0.25, 0.82)	0.297	0.07 (−1.56, 1.69)	0.937
DOM vs. NDOM	0.30 (−0.26, 0.87)	0.294	0.12 (−1.66, 1.89)	0.897
Speed × IPSI	0.88 (−0.48, 2.24)	0.202	0.30 (−2.27, 2.88)	0.817
Speed × DOM	0.71 (−0.69, 2.10)	0.321	0.50 (−2.08, 3.07)	0.704
IPSI × DOM	−1.01 (−2.12, 0.10)	0.075	0.15 (−3.03, 3.32)	0.927
Speed × IPSI × DOM	−1.36 (−3.92, 1.20)	0.297	−1.16 (−6.43, 4.11)	0.667

IPSI = foot ipsilateral to the affected shoulder; CON = contralateral foot; DOM = dominant foot; NDOM = non-dominant foot.

### 3.2 Affected-side effects of internal foot tissue stresses and modulation by walking speed and dominance

Model-estimated marginal means of internal foot tissue stresses under different walking speeds and foot attribute combinations are presented in Table 6. Overall, as walking speed increased from the preferred to the critical level, stresses associated with the push-off phase—including forefoot soft tissue stress and plantar fascia stress—consistently exhibited an increasing trend, whereas changes in heel soft tissue stress during the loading response phase were relatively small. Stress distributions across different foot attribute combinations showed substantial overlap, and no clearly delineated pattern of affected-side laterality was observed.

**Table 6:** Model-estimated marginal means (EMMs) of heel soft tissue stress, forefoot soft tissue stress, and plantar fascia stress under different walking speeds and foot attribute combinations.

Combination	Speed (km/h)	Heel stress (MPa)	Forefoot stress (MPa)	Fascia stress (MPa)
CON–DOM	5	0.163 (0.153, 0.174)	0.251 (0.226, 0.276)	29.68 (26.37, 32.99)
CON–NDOM		0.154 (0.144, 0.164)	0.248 (0.206, 0.289)	29.08 (24.37, 33.80)
IPSI–DOM		0.145 (0.128, 0.163)	0.256 (0.220, 0.291)	30.06 (25.76, 34.36)
IPSI–NDOM		0.163 (0.152, 0.174)	0.252 (0.231, 0.273)	29.57 (26.95, 32.19)
CON–DOM	7	0.182 (0.166, 0.198)	0.296 (0.278, 0.313)	36.43 (33.48, 39.39)
CON–NDOM		0.160 (0.122, 0.198)	0.288 (0.278, 0.297)	34.44 (32.51, 36.37)
IPSI–DOM		0.156 (0.114, 0.198)	0.286 (0.275, 0.297)	34.24 (32.20, 36.27)
IPSI–NDOM		0.185 (0.168, 0.202)	0.293 (0.277, 0.309)	35.76 (32.84, 38.68)

IPSI = foot ipsilateral to the affected shoulder; CON = contralateral foot; DOM = dominant foot; NDOM = non-dominant foot.

As an effect-size metric reflecting the magnitude and direction of laterality, affected-side symmetry indices (SIs) were further derived from the model-estimated marginal means (Table 7). The results indicated that heel soft tissue stress exhibited a mild negative laterality under preferred-speed conditions, whereas this laterality was attenuated and approached zero at the critical speed. Symmetry indices for forefoot soft tissue stress and plantar fascia stress were small under both speed conditions, indicating that internal tissue stresses during the push-off phase remained largely symmetrical between the two sides.

**Table 7:** Affected-side laterality of internal foot tissue stresses derived from model-estimated marginal means (SI, %).

Outcome	Speed (km/h)	SI (IPSI vs. CON), %
Heel stress	5	−3.7 (−9.6, 2.2)
	7	−1.9 (−8.8, 5.0)
Forefoot stress	5	1.2 (−6.4, 8.9)
	7	−1.0 (−5.9, 3.8)
Fascia stress	5	0.9 (−7.3, 9.1)
	7	−2.3 (−8.7, 4.1)

Statistical inference results from the foot-level linear mixed-effects models are presented in Table 8. For heel soft tissue stress during the loading response phase, the main effect of affected-side status did not reach statistical significance (*p* = 0.219). The main effect of walking speed was likewise non-significant, and the modulatory effect of speed on affected-side differences also failed to reach statistical significance (*p* = 0.314). The interaction between affected-side status and dominance status exhibited a marginal trend (*p* = 0.124), suggesting that affected-side stress distribution may be influenced by functional foot dominance; however, this tendency did not constitute robust statistical evidence given the current sample size. For forefoot soft tissue stress during the push-off phase, the main effect of walking speed was statistically significant (*p* = 0.025), indicating a substantial increase in propulsion-related internal tissue loading under critical-speed conditions. In contrast, the main effect of affected-side status, the interaction between walking speed and affected-side status, and interaction terms involving dominance status were all non-significant (all *p* > 0.8). Plantar fascia stress similarly demonstrated a significant main effect of walking speed (*p* = 0.003), whereas the main effect of affected-side status and its interactions with walking speed and dominance status did not reach statistical significance (all *p* > 0.5).

**Table 8:** Foot-level mixed-effects model results for internal foot tissue stresses (speed × IPSI × DOM).

Fixed effect	Heel stress		Forefoot stress		Plantar fascia stress	
	*β* (95% CI)	*p*	*β* (95% CI)	*p*	*β* (95% CI)	*p*
Speed (7 vs. 5 km/h)	0.0058 (−0.0228, 0.0344)	0.691	0.0400 (0.0050, 0.0751)	0.025	5.3608 (1.7759, 8.9456)	0.003
IPSI vs. CON	0.0091 (−0.0054, 0.0237)	0.219	0.0044 (−0.0422, 0.0510)	0.853	0.4874 (−4.9062, 5.8810)	0.859
DOM vs. NDOM	0.0092 (−0.0049, 0.0234)	0.200	0.0033 (−0.0451, 0.0518)	0.893	0.5974 (−5.1633, 6.3580)	0.839
Speed × IPSI	0.0160 (−0.0151, 0.0471)	0.314	0.0011 (−0.0460, 0.0482)	0.964	0.8330 (−5.0098, 6.6758)	0.780
Speed × DOM	0.0127 (−0.0191, 0.0445)	0.433	0.0048 (−0.0436, 0.0532)	0.846	1.3943 (−4.4294, 7.2179)	0.639
IPSI × DOM	−0.0270 (−0.0613, 0.0074)	0.124	0.0002 (−0.0897, 0.0902)	0.996	−0.1058 (−10.8224, 10.6109)	0.985
Speed × IPSI × DOM	−0.0243 (−0.0842, 0.0357)	0.428	−0.0156 (−0.1109, 0.0798)	0.749	−3.4128 (−15.3434, 8.5179)	0.575

IPSI = foot ipsilateral to the affected shoulder; CON = contralateral foot; DOM = dominant foot; NDOM = non-dominant foot.

## 4 Discussion

This study systematically examined affected-side laterality in plantar external loading and internal foot tissue stresses in individuals with unilateral shoulder dislocation during preferred-speed and critical-speed walking, as well as their responses to walking speed as an external challenge. The results demonstrated that, under both speed conditions, rearfoot and forefoot peak forces remained largely symmetrical between the foot ipsilateral to the affected shoulder and the contralateral foot, with small symmetry index magnitudes. Consistently, the linear mixed-effects models did not detect significant main effects of affected-side status or significant interactions between affected-side status and walking speed. Finite element analyses further revealed that, although increasing walking speed significantly elevated internal stress levels in forefoot soft tissues and the plantar fascia during the push-off phase, the mechanical responses of key foot tissues did not exhibit a clear affected-side–dependent distribution. Overall, walking speed as an external challenge primarily amplified the absolute level of foot mechanical loading, rather than systematically enhancing mechanical asymmetry between the ipsilateral and contralateral feet at the statistical level. Therefore, the initial hypothesis of lateralized distal compensation was not supported. Instead, the observed pattern was more consistent with bilateral, speed-related load modulation. Importantly, because no healthy control group or longitudinal pre-injury comparison was included, this bilateral response should not be interpreted as evidence of a shoulder-dislocation-specific distal adaptation.

### 4.1 Hierarchical distribution of shoulder dislocation–related compensation and the absence of foot-level laterality

Previous studies have shown that restricted or asymmetric arm swing can influence overall gait control by altering trunk rotation patterns and whole-body angular momentum regulation strategies (1, 2, 20, 21). However, the present findings indicate that such control strategy alterations induced by unilateral shoulder dislocation did not translate into pronounced affected-side laterality in plantar external loading or internal foot tissue stresses during level-ground walking. One plausible explanation is that walking, as a highly redundant and stability-prioritized cyclic motor task (22–24), does not necessarily amplify compensatory mechanisms progressively along the longitudinal axis of the body. One possible interpretation is that altered upper-limb or trunk-control strategies, if present, may not necessarily propagate to the foot as measurable affected-side loading differences during level treadmill walking. However, because arm swing, trunk rotation, pelvic motion, lower-limb joint kinematics, and electromyography were not directly measured, this interpretation remains speculative and should be tested in future studies.

Compared with high-impact tasks such as running or jumping, walking is characterized by a pronounced double-support phase (25), and both central pattern generation and energy-efficiency optimization mechanisms favor maintaining a high degree of bilateral symmetry in lower-limb output (26, 27). Within this control context, even when lateralized perturbations exist at the level of the upper limbs or trunk, lower-limb—and particularly foot–ground contact—mechanical outputs may remain constrained within a relatively narrow symmetrical range. The present results showed that symmetry indices for both rearfoot impact peak forces and forefoot push-off peak forces remained low, supporting the notion that the foot is not the most sensitive distal “readout” of altered upper-limb control. Although increasing walking speed significantly elevated propulsion-related external loads and internal tissue stress levels, this speed challenge did not systematically amplify affected-side laterality. This finding further suggests that, under the conditions examined, walking speed primarily modulated the absolute magnitude of loading rather than the bilateral distribution pattern. At the critical speed, participants maintained continuous walking without entering a flight phase, and their control strategy remained centered on stability preservation. In this context, the neuromuscular system is more likely to respond to increased mechanical demands through bilateral, concordant load elevation rather than adopting lateralized loading strategies, thereby minimizing the risk of dynamic instability.

### 4.2 Effects of walking speed as an external challenge on foot loading magnitude and distribution patterns

Although the results demonstrated clear and consistent effects of increased walking speed on foot mechanics, these effects were primarily reflected in elevations of absolute loading and tissue stress levels rather than in a redistribution between the affected and contralateral sides. At both the plantar external loading and internal tissue stress levels, the main effect of speed manifested as significant increases in push-off–related metrics, with particularly pronounced and concurrent elevations in forefoot peak force, forefoot soft tissue stress, and plantar fascia stress. These findings reflect an overall enhancement of propulsive demand and energy output at higher walking speeds. Such results are consistent with previous studies on the relationship between walking speed and propulsion dynamics (28–30), and confirm that the critical-speed condition employed in the present study constituted an effective external challenge from a mechanical perspective. Contrary to the initial hypothesis, however, increasing walking speed did not amplify affected-side laterality associated with shoulder dislocation. Neither symmetry indices derived from external loading measures nor finite element–estimated stresses in key foot tissues exhibited statistically significant interactions between walking speed and affected-side status. This pattern suggests that, during level-ground walking as examined here, the primary strategy for accommodating speed-related challenges is a bilateral and coordinated elevation of loading to meet increased propulsion and stability demands, rather than the adoption of lateralized mechanical distributions. In other words, the speed challenge altered “how much load” the system experienced, without fundamentally changing “which side bore more load.”

From a motor control perspective, this finding is intrinsically plausible. Although the critical speed approached the boundary between walking and running, participants maintained a continuous walking pattern without a flight phase, and the underlying neural control mechanisms and dynamical organization remained characteristic of walking. Within this control framework, introducing pronounced asymmetries in loading could increase the risk of dynamic instability; consequently, the neuromuscular system appears more inclined to adopt a bilaterally concordant amplification of loading to accommodate escalating mechanical demands. Taken together, these observations indicate that walking speed per se does not necessarily elicit distal lateralized compensation, and that its effects are strongly constrained by the specific task context and the associated control mode.

### 4.3 Dissociation between external load symmetry and internal tissue mechanical responses

Notably, while plantar external loading remained highly symmetrical between the two sides, stress levels in key internal foot soft tissues increased significantly with walking speed. This finding suggests that distal compensation does not necessarily manifest as asymmetry, but may instead be reflected by an overall upward shift in the mechanical environment experienced by internal foot tissues under conditions where external loading patterns remain symmetrical. Consequently, assessments based solely on plantar external loads or symmetry indices may be insufficient to fully capture the potential mechanical costs at the tissue level.

From a mechanical standpoint, plantar external loading represents the net reaction arising from foot–ground interactions, whereas internal tissue stresses are jointly determined by local geometry, material properties, and load transmission pathways within the foot (16, 31, 32). As walking speed increases, even when peak force magnitudes remain comparable between the two feet, structures such as the forefoot soft tissues and the plantar fascia are required to accommodate higher propulsion-related loads over shorter time scales, thereby increasing the likelihood of internal stress concentration and elevated peak stresses. Finite element analyses in the present study indicated that forefoot soft tissue and plantar fascia stresses were particularly sensitive to changes in walking speed. However, this sensitivity was not accompanied by pronounced affected-side laterality. These findings provide important insights into the distal consequences of shoulder dislocation. Although alterations in upper-limb control strategies did not manifest as overtly asymmetric load distribution at the foot level, foot tissues were nonetheless subjected to an overall increase in mechanical demand under higher walking speeds or elevated functional requirements. Such a “symmetrical but intensified” loading pattern may, under conditions of prolonged or repeated exposure, increase the potential risk of soft tissue overload in the foot, independent of the presence of affected-side laterality.

In this study, experimentally derived region-specific peak forces were translated into internal foot tissue stress distributions using a finite element model, providing a feasible pathway to bridge gait experimental findings with tissue-level mechanical interpretations. Although this loading approach involved appropriate simplifications with respect to temporal profiles and load directions, the resulting estimates were able to capture the relative mechanical effects of changes in external loading on key soft tissues during specific functional phases, namely loading response related stability and push-off related propulsion. Accordingly, the finite element analysis in this study was not intended to predict absolute stress thresholds for tissue failure, but rather to serve as a mechanistic analytical tool to elucidate how the internal mechanical environment of the foot undergoes systematic changes in response to task demands, even when external loading appears symmetrical.

### 4.4 Potential modulatory role of foot dominance in distal mechanical responses

Previous studies have suggested that the dominant and non-dominant feet are not entirely equivalent in terms of motor control strategies and functional roles (33). The dominant foot is typically more involved in tasks requiring fine control or propulsion, whereas the non-dominant foot contributes more to postural stability and support. Accordingly, when unilateral alterations in upper-limb control are present, compensatory strategies may not be governed solely by left–right anatomical symmetry, but may instead incorporate functional foot attributes to subtly adjust load distribution patterns. In the present study, interactions involving affected-side status and foot dominance showed only non-significant tendencies for selected mechanical outcomes and did not exhibit consistent directional patterns across plantar external loading and FE-estimated internal tissue stresses. Given the small sample size and uneven distribution of affected-side and dominant-foot combinations, these findings should be interpreted cautiously. Although these effects did not reach conventional levels of statistical significance and did not display consistent directional patterns across different mechanical metrics, they nonetheless provide informative clues for understanding inter-individual variability in distal compensation strategies.

It should be noted that this modulatory effect did not constitute robust statistical evidence in the current sample, which may be attributable to the relatively small sample size, the uneven individual-level combinations of affected-side and dominance-side status, and the strong constraints imposed by the walking task itself toward bilaterally symmetrical output.

### 4.5 Limitations, clinical implications, and future directions

Several limitations of this study should be acknowledged. First, the sample size was relatively small, and among the participants ultimately included in the analysis, the individual-level combinations of affected-side status and foot dominance were unevenly distributed. This imbalance may have reduced the statistical power to detect affected-side laterality and associated interaction effects. In addition, the limited final sample size constrained the use of more complex random-effects structures and reduced the statistical power for detecting interaction effects. To partially offset this, foot-level outcomes were analyzed using linear mixed-effects models that preserved the within-participant data structure and made full use of the available observations, and individualized finite element models were used for mechanism-oriented, relative comparisons. Although 30 individuals were initially recruited, only 12 participants completed both walking-speed conditions and were included in the final analysis. The final sample therefore represents participants who could tolerate relatively fast treadmill walking, rather than the broader population of individuals with unilateral shoulder dislocation. And, detailed clinical characteristics, such as standardized shoulder function scores, kinesiophobia scores, and detailed pain responses during testing, were not available for all participants. Moreover, the bilateral symmetry was supported not only by non-significant inferential tests but also by consistently small symmetry-index magnitudes across multiple external and internal measures, reducing the likelihood that it merely reflects insufficient statistical power. Second, under the critical-speed condition, participants maintained a continuous walking pattern without transitioning to running or exhibiting a flight phase. This constraint likely limited the extent to which the speed challenge perturbed motor control strategies, potentially obscuring distal lateralized compensations that might emerge under higher functional demands or more unstable task conditions. In addition, as described in the Methods, the finite element modeling of the foot involved necessary simplifications; accordingly, the resulting estimates are intended primarily for relative, mechanism-oriented comparisons rather than for determining absolute stress thresholds or directly inferring tissue injury risk.

From a preliminary clinical perspective, the present findings do not provide evidence of marked affected-side foot-loading asymmetry under the tested treadmill-walking conditions. However, faster walking was associated with higher forefoot loading and higher FE-estimated forefoot soft-tissue and plantar fascia stresses. These results suggest that load progression during faster walking may deserve attention during rehabilitation and physical reconditioning. Nevertheless, this implication should be interpreted cautiously because the study did not include a healthy control group, long-term follow-up, or foot pain and injury outcomes.

Future studies may further investigate distal compensation mechanisms associated with shoulder dislocation under more challenging motor tasks, such as running, directional changes, asymmetric loading, or externally imposed perturbations. Integrating these paradigms with kinematic or electromyographic analyses of the upper limbs and trunk could provide deeper insight into compensation transmission pathways across different control levels and their long-term implications for the mechanical environment of distal tissues.

## 5 Conclusion

By integrating plantar pressure measurements with foot finite element analysis, this study systematically examined affected-side laterality in external foot loading and internal tissue stresses in individuals with unilateral shoulder dislocation during preferred-speed and critical-speed walking, as well as their responses to walking speed as an external challenge. The findings demonstrated that, during level-ground walking, rearfoot and forefoot peak forces and stresses in key internal foot soft tissues remained largely symmetrical between the foot ipsilateral to the affected shoulder and the contralateral foot. Increases in walking speed were primarily associated with overall elevations in propulsion-related loads and tissue stress levels, rather than with a systematic amplification of affected-side laterality. These findings suggest that, under the tested conditions, speed-related changes were expressed primarily as bilateral increases in mechanical demand rather than side-specific load redistribution. Because no healthy control group or longitudinal comparison was included, the results should be interpreted as preliminary within-cohort observations rather than evidence of shoulder-dislocation-specific distal adaptation. From a tissue-mechanics perspective, the present study provides new evidence for understanding distal biomechanical consequences of proximal injury and establishes a foundation for future investigations of distal compensation mechanisms under more challenging motor tasks. Because this study did not include a healthy control group or conduct longitudinal comparisons, the results should be interpreted with caution.

## Data Availability

The datasets presented in this article are not readily available because the de-identified data supporting the conclusions of this article will be made available by the corresponding author upon reasonable request, subject to institutional ethics and privacy restrictions. Requests to access the datasets should be directed to xiaoliufeng@tmmu.edu.cn.
